# Facility-Level Variation in Dialysis Use and Mortality Among Older Veterans With Incident Kidney Failure

**DOI:** 10.1001/jamanetworkopen.2020.34084

**Published:** 2021-01-15

**Authors:** Christina Bradshaw, I-Chun Thomas, Maria E. Montez-Rath, Karl A. Lorenz, Steven M. Asch, John T. Leppert, Virginia Wang, Ann M. O’Hare, Manjula Kurella Tamura

**Affiliations:** 1 Division of Nephrology, Department of Medicine, Stanford University School of Medicine, Palo Alto, California; 2 Geriatric Research and Education Clinical Center, Veterans Affairs (VA) Palo Alto Health Care System, Palo Alto, California; 3 Center for Innovation to Implementation, VA Palo Alto Health Care System, Palo Alto, California; 4 Primary Care and Population Health, Stanford University School of Medicine, Palo Alto, California; 5 Division of Urology, VA Palo Alto Health Care System, Palo Alto, California; 6 Department of Urology, Stanford University School of Medicine, Palo Alto, California; 7 Center of Innovation for Health Services Research, Durham VA Health Care System, Durham, North Carolina; 8 Department of Population Health Sciences, Duke University School of Medicine, Durham, North Carolina; 9 Division of General Internal Medicine, Department of Medicine, Duke University School of Medicine, Durham, North Carolina; 10 Hospital and Specialty Medicine Service, VA Puget Sound Health Care System, Seattle, Washington; 11 Division of Nephrology, Department of Medicine, University of Washington School of Medicine, Seattle

## Abstract

**Importance:**

Current guidelines lack consensus regarding the treatment of patients who may not benefit from dialysis; this lack of consensus may be associated with the substantial variation in dialysis use and outcomes across health care facilities.

**Objective:**

To assess the degree to which variation in dialysis use and mortality was associated with patient rather than facility characteristics and to distinguish which features identified the US Department of Veterans Affairs (VA) facilities with high rates of dialysis use.

**Design, Setting, and Participants:**

This cohort study analyzed data of veterans with stage 3 or 4 chronic kidney disease that progressed to kidney failure between January 1, 2011, and December 31, 2014. These patients received care from VA facilities across the US. Data sources included laboratory and administrative records from the VA, Medicare, and United States Renal Data System. Data analysis was conducted from August 1, 2019, to September 1, 2020.

**Exposures:**

The primary exposure was the VA facility in which patients received most of their care before the onset of incident kidney failure defined as the first occurrence of either a sustained estimated glomerular filtration rate of less than 15 mL/min/1.73 m^2^ or the initiation of maintenance dialysis.

**Main Outcomes and Measures:**

The primary outcomes were dialysis use and mortality within 2 years of incident kidney failure. Median rate ratio was used to quantify facility-level variation, and variance partition coefficient was used to quantify the sources of unexplained variation.

**Results:**

The cohort included 8695 older veterans with a mean (SD) age of 78.8 (7.5) years who were predominantly male (8573 [99%]) and White (6102 [70%]) individuals treated at 108 VA facilities. The observed frequency of dialysis use across facilities ranged from 25.0% to 81.4%, with a median (interquartile range [IQR]) rate of 51.7% (48.4%-60.0%). The observed frequency of mortality across facilities ranged from 27.2% to 60.0%, with a median (IQR) rate of 45.2% (41.2%-48.6%). The median rate ratio (adjusted for multiple patient and facility characteristics) was 1.40 for dialysis use and 1.08 for mortality. The unexplained variation in both outcomes mainly derived from patient characteristics rather than facility characteristics. No correlation was found between dialysis use and mortality at the facility level (correlation coefficient = 0.03).

**Conclusions and Relevance:**

This study found sizable variation in dialysis use for older adults that was poorly correlated with facility-level mortality rates and was not accounted for by differences in measured patient and facility characteristics. These findings suggest opportunities to improve the degree to which dialysis use practices align with the values, goals, and preferences of older adults with kidney failure.

## Key Points

**Question:**

To what extent do dialysis use and mortality vary among older adults with incident kidney failure, and are these variations associated with patient or facility factors?

**Findings:**

In this cohort study of 8695 older adults with incident kidney failure, dialysis use varied widely across Veterans Affairs facilities with minimal variation in mortality. Most of the variation was associated with patient characteristics, and no correlation was found between the facility-level rate of dialysis use and mortality.

**Meaning:**

Results of this study suggest that there is marked variation in dialysis use practices for older adults across Veterans Affairs facilities.

## Teaser

This cohort study examines which patient and facility factors in the US Department of Veterans Affairs health care system are associated with dialysis approaches and outcomes.

## Introduction

For older adults approaching kidney failure, the decisions about whether and when to initiate maintenance dialysis are often complex given that the benefits of dialysis become less certain with increasing age and comorbidity burden.^1,2,3^ Guidelines, therefore, recommend the consideration of older individuals’ unique clinical histories and preferences when selecting treatment.^4^ Previous studies have observed variations in dialysis use across geographic regions and health care systems, suggesting that structural and practice-related factors affect their treatment.^5,6,7,8^ However, these comparisons are likely confounded by differences in the prevalence of chronic kidney disease (CKD) and other case-mix factors across settings.

Evaluating variation in dialysis use and mortality at the facility level could provide insights into dialysis practices as well as inform policy and quality improvement efforts, which are often undertaken at the facility level. Improved understanding of the degree and sources of variation within a health care system can also help to identify best practices when treating older adults and to identify policy and practice levers that might be targeted for quality improvement initiatives.

Therefore, we sought to quantify variation in dialysis use and mortality among older adults who progressed to kidney failure within the national US Department of Veterans Affairs (VA) integrated health care system. We assessed the degree to which variation was associated with patient rather than facility characteristics, and we distinguished which features identified the VA facilities with high rates of dialysis use. We also evaluated whether the facility-level rate of dialysis use had a correlation with mortality.

## Methods

This retrospective cohort study was approved by the Stanford University Institutional Review Board and the VA Palo Alto Health Care System Office of Research and Development. The Stanford University Institutional Review Board waived the requirement for informed consent because the study used data that had already been collected and, therefore, the study was considered to have minimal risk to individuals. We followed the Strengthening the Reporting of Observational Studies in Epidemiology (STROBE) reporting guideline.

### Study Population

Using laboratory and administrative data from the VA, Medicare, and United States Renal Data System (USRDS), we identified a cohort of veterans aged 67 years or older with stage 3 or 4 CKD who progressed to kidney failure between January 1, 2011, and December 31, 2014. Consistent with the Kidney Disease Improving Global Outcomes classification, we defined incident kidney failure as the first occurrence of either a sustained estimated glomerular filtration rate (eGFR) less than 15 mL/min/1.73 m^2^ (using the Chronic Kidney Disease Epidemiology Collaboration equation) or the initiation of maintenance dialysis.^9,10^ To meet the eGFR criteria for incident kidney failure, we required 2 eGFR measurements of less than 15 mL/min/1.73 m^2^ taken at least 5 days apart, and 1 of these measurements had to be taken during an outpatient visit. We restricted the analyses to veterans who had at least 1 inpatient or outpatient visit at a VA facility during the previous year.

We excluded individuals if they had no eGFR measurements or had received nephrology care (as recorded in Medicare claims data) in the previous year because decisions about dialysis for these patients likely occurred outside the VA. We also excluded individuals with an inconsistent date of death and those with acute kidney injury resulting in kidney failure, which was defined as an eGFR greater than 30 mL/min/1.73 m^2^ in the previous month. After excluding patients from low-volume VA facilities and facilities outside of the 48 contiguous states, the final cohort consisted of 8695 veterans from 108 VA facilities (eFigure in the Supplement).

### Exposure, Outcomes, and Covariates

The primary exposure for this study was the VA facility in which patients received most of their care before developing kidney failure. Eligible VA facilities were identified through the VA Tracking System, which defines a VA facility as providing at least 2 categories of care (inpatient, outpatient, residential, or extended care). The exposure facility could include one that did not have on-site outpatient dialysis services.

Patients were followed up from the index date (the first time they developed kidney failure) to up to 2 years for each outcome. The primary outcome was dialysis use within 2 years of the index date. Dialysis use was defined as the presence of a dialysis start date recorded in the USRDS or at least 1 outpatient dialysis procedure code recorded in VA administrative files or Medicare claims. To identify dialysis procedures in the VA or Medicare data, we used the *International Classification of Diseases, Ninth Revision* (*ICD-9*) and *International Statistical Classification of Diseases and Related Health Problems, Tenth Revision* (*ICD-10*) codes 3995, 5498, V56, V56.8, V45.1, Z99.2, and Z49 as well as *Current Procedural Terminology* codes 909.35, 909.37, and 909.47. We ascertained death within 2 years of the index date using the VA Vital Status File.

We used USRDS, VA, and Medicare data to define covariates at the facility and patient levels. Facility characteristics included facility complexity, Veterans Integrated Service Network region, and presence of an outpatient dialysis unit. We obtained information on VA dialysis facility capacity and occupancy from the Veterans Health Administration National Kidney Program and VA Renal SharePoint site as a proxy for the supply of dialysis resources in a given VA facility.^11^ Patient characteristics included age at the time of incident kidney failure, sex, race/ethnicity, metropolitan vs nonmetropolitan residence based on Rural-Urban Continuum codes, driving distance to the nearest VA facility, co-payment for VA services, hospitalizations, nephrology care in the previous year, and comorbidities. Using the method described by Quan et al,^12^ we ascertained comorbidities over the 2-year period preceding the index date of kidney failure using *ICD-9* and *ICD-10* codes from VA and Medicare claims for diabetes, ischemic heart disease, peripheral vascular disease, cerebrovascular disease, congestive heart failure, cancer, chronic lung disease, liver disease, dementia, depression, posttraumatic stress disorder, paralysis, and rheumatologic disease.

### Statistical Analysis

We examined baseline facility and patient characteristics for the analytic cohort, expressing categorical variables as numbers (percentages) and continuous variables as mean (SD) or median (interquartile range [IQR]) values, as appropriate. For each facility, we calculated the 2-year rate of dialysis use and the 2-year total mortality rate. We then calculated the observed median 2-year frequency of dialysis use and mortality across facilities.

To quantify variation across VA facilities, we estimated the median rate ratio (MRR) of dialysis use and total mortality. The MRR expresses the median relative difference in the rate of an outcome between an individual in a facility with a higher rate of the outcome and an identical individual in a facility with a lower rate of the outcome.^13^ Values start at 1, which corresponds with no facility-level variation in an outcome. The higher the MRR, the larger the variation between facilities. For example, an MRR of 1.40 for dialysis use means that, for 50% of possible pairwise comparisons of identical individuals, the rate of dialysis use is approximately 40% greater when comparing a facility with higher dialysis use to a facility with lower dialysis use.

To estimate MRRs, we constructed multilevel Poisson regression models with an offset for time at risk during the 2 years after the index date of incident kidney failure.^14^ Three multilevel models were constructed for each outcome. The base model included a random intercept for the facility and no covariates. The second model was adjusted for patient covariates, and the third model was adjusted for patient and facility covariates; we accounted for clustering of patients within facilities. Data were missing for patient-level variables only; the percentage of missing values was low (<1%) for most variables, with the exception of serum albumin (9%). We assumed data were missing at random and conditional on observed variables. We handled missing data using multiple imputation, and we applied Rubin rules to combine the results.^15,16,17^ Patient characteristics other than age, sex, and race/ethnicity were included in models after selection according to the following algorithm: (1) variables without missing data were selected if univariate *P* values were smaller than *P* = .20, and (2) variables with missing data were selected if the *P* value from a univariate model run on multiple imputed data sets was *P* < .20 ninety percent of the time.^15^ In sensitivity analyses, we computed MRRs for dialysis use and death among patients 80 years or older and in the subset of the cohort who first met the eGFR less than 15 mL/min/1.73 m^2^ criterion for kidney failure (n = 7225); we excluded individuals who started dialysis before progressing to an eGFR below this level.

To elucidate the sources of variation, we used the multilevel model to calculate the variance partition coefficient (VPC). The VPC quantifies the proportion of unexplained variation in an outcome that is attributable to between-facility differences after adjusting for all patient- and facility-level factors included in the model.^13,14^ Values range from 0 to 1; a VPC closer to zero indicates that most of the unexplained variation in an outcome is attributed to within-facility and between-patient differences rather than between-facility differences. Given that the VPC from a Poisson model depends on the included covariates, we chose to calculate the VPC for a referent individual at different age groups because we hypothesized that age was the most important factor of variation in the outcomes.

Using the patient-level models, we computed each facility’s expected frequency of dialysis use and expected frequency of deaths. We identified facility and patient characteristics that were associated with high (upper 90th percentile of the expected frequency of dialysis use), medium (10th to 90th percentile), and low (lower 10th percentile) frequency of dialysis use. We also used the Pearson correlation coefficient to assess the association between dialysis use and mortality at the facility level.

All tests were 2-tailed and unpaired and were performed at the *P* = .05 significance level. Statistical analyses were performed using a combination of SAS, version 9.4 (SAS Institute Inc), and Stata/MP, version 15.1 (StataCorp LLC). Data analysis was conducted from August 1, 2019, to September 1, 2020.

## Results

The cohort of 8695 veterans with incident kidney failure had a mean (SD) age of 78.8 (7.5) years, included predominantly male (8573 [99%]) and White (6102 [70%]) individuals, and resided in metropolitan areas (Table 1). The most common comorbidities were diabetes (5410 [62%]), ischemic heart disease (4252 [49%]), congestive heart failure (3527 [41%]), and chronic lung disease (2975 [34%]); approximately 9% of these patients (n = 787) were nursing home residents. At the time of incident kidney failure, 7376 individuals (85%) had an eGFR of 10 mL/min/1.73 m^2^ or higher and 4777 (55%) had seen a nephrologist at least twice in the preceding year.

**Table 1.** Baseline Facility and Patient Characteristics

Variable	Overall, No. (%)
**Facility characteristics**	
Facilities, No.	108
Facility complexity	
High	74 (69)
Medium/low	34 (32)
VISN region	
West	24 (22)
Northeast	20 (19)
Southeast	38 (35)
Midwest	26 (24)
Outpatient dialysis unit	65 (60)
Maximum capacity, median (IQR)	52.0 (40.0 to 66.0)
% Occupancy, median (IQR)	90.9 (76.4 to 97.5)
**Patient characteristics**	
Patients, No.	8695
Demographic	
Age at incident kidney failure, mean (SD), y	78.8 (7.5)
Age group, y	
67 to <70	1406 (16)
70 to <75	1661 (19)
75 to <80	1749 (20)
80 to <85	1842 (21)
≥85	2037 (23)
Male sex	8573 (99)
Race/ethnicity	
White	6102 (70)
Black	1853 (21)
Other^a^	731 (8)
Missing	9 (0)
Residence	
Metropolitan	6685 (77)
Nonmetropolitan	2007 (23)
Zip code median income, US $	
<41 500	2938 (34)
41 500 to <54 000	2702 (31)
≥54 000	2933 (34)
Missing	122 (1)
Driving distance to nearest VA facility, median (IQR), miles	28.2 (11.3 to 61.3)
Co-payment for VA services	6620 (76)
Missing	145 (2)
Medical history	
Comorbidities	
Diabetes	5410 (62)
Ischemic heart disease	4252 (49)
Peripheral vascular disease	2637 (30)
Cerebrovascular disease	2077 (24)
Congestive heart failure	3527 (41)
Cancer	2429 (28)
Chronic lung disease	2975 (34)
Liver disease	467 (5)
Dementia	812 (9)
Paralysis	153 (2)
Depression/PTSD	1824 (21)
Rheumatologic disease	218 (3)
Nursing home resident	787 (9)
Hospitalizations in previous year, median (IQR)	0.0 (0.0 to 1.0)
Serum albumin level, mg/dL	
≥3.5	4731 (54)
<3.5	3054 (35)
Missing	910 (11)
eGFR at incident kidney failure, mL/min/1.73 m^2^	
≥10	7376 (85)
<10	1258 (15)
Missing	61 (1)
Rate of eGFR decline before incident kidney failure, median (IQR), mL/min/y	−3.6 (−6.0 to −2.0)
Missing	99 (1)
Pre–kidney failure nephrology visits	
None	3245 (37)
1	673 (8)
≥2	4777 (55)

Abbreviations: eGFR, estimated glomerular filtration rate; IQR, interquartile range; PTSD, posttraumatic stress disorder; VA, US Department of Veterans Affairs; VISN, Veterans Integrated Service Network.

SI conversion factors: To convert miles to kilometers, multiply by 1.6; serum albumin to grams per liter, multiply by 10.

^a^ Other includes Pacific Islander, Asian, and American Indian.

A total of 108 VA facilities were included, most of which were high-complexity facilities (74 [69%]) and had an outpatient dialysis unit (65 [60%]) (Table 1). The median (IQR) number of patients with incident kidney failure per facility over the 2-year follow-up period was 74 (45-106).

### Variation in Dialysis Use

Across the 108 facilities, the observed frequency of dialysis use ranged from 25.0% to 81.4%, with a median (IQR) rate of 51.7% (48.4%-60.0%). Among patients who started dialysis, 3895 (83%) started hemodialysis rather than peritoneal dialysis, 2833 (73%) of whom started hemodialysis with a catheter. The unadjusted MRR for dialysis use was 1.35, and the fully adjusted MRR, including facility and patient characteristics, was 1.40 (Table 2). Compared with younger individuals, those 80 years or older had slightly more variation in dialysis use (fully adjusted MRR, 1.45 vs 1.35). In sensitivity analyses restricted to the cohort who met the eGFR criterion of less than 15 mL/min/1.73 m^2^ for incident kidney failure, the fully adjusted MRR for dialysis was 1.23 (Table 2). The fully adjusted VPC was 0.27 for individuals aged 80 to 84 years and 0.18 for those 85 years or older. The VPC was larger at younger ages, indicating that the unexplained variation in dialysis use at older ages was more likely derived from between-patient differences than the variation found at younger ages.

**Table 2.** Facility-Level Median Rate Ratios for Dialysis Use and Mortality

Model	Median rate ratio			
	Full cohort (N = 8695)		eGFR <15 mL/min/1.73 m^2^ cohort (n = 7225)	
	Dialysis use	Mortality	Dialysis use	Mortality
Unadjusted	1.35	1.05	1.17	1.05
Adjusted for patient characteristics	1.42^a^	1.11^b^	1.25	1.12
Fully adjusted	1.40^c^	1.08^d^	1.23	1.08

Abbreviation: eGFR, estimated glomerular filtration rate.

^a^ Models were adjusted for patient age, sex, race/ethnicity, driving distance to the nearest US Department of Veterans Affairs (VA) facility, serum albumin level, eGFR at incident kidney failure, rate of eGFR decline, nephrology care, and the following comorbidities: cancer, chronic lung disease, liver disease, congestive heart failure, dementia, diabetes, ischemic heart disease, and peripheral vascular disease.

^b^ Models were adjusted for patient age, sex, race/ethnicity, driving distance to the nearest VA facility, co-payment, serum albumin level, eGFR at incident kidney failure, rate of eGFR decline, nephrology care, and the following comorbidities: cancer, chronic lung disease, liver disease, congestive heart failure, dementia, paralysis, ischemic heart disease, peripheral vascular disease, cerebrovascular disease, rheumatologic disease, nursing home residence, and number of hospitalizations in the year before incident kidney failure.

^c^ Models were adjusted for patient characteristics cited in note a and the following facility characteristics: facility complexity, Veterans Integrated Service Network (VISN) region, presence of outpatient dialysis unit, maximum dialysis unit capacity, and percent occupancy of dialysis unit.

^d^ Models were adjusted for patient characteristics cited in note b and the following facility characteristics: facility complexity, VISN region, presence of outpatient dialysis unit, maximum dialysis unit capacity, and percent occupancy of dialysis unit.

Figure, A shows the distribution of expected dialysis use by facility, adjusted for patient characteristics. There were no significant differences in facility characteristics between high-use facilities and low- or medium-use facilities. However, compared with low- or medium-use facilities, high-use facilities were less likely to serve patients from zip codes with the highest median income (23% vs 40% and 34%) and more likely to serve patients who did not receive nephrology care in the previous year (41% vs 36% and 49%). Compared with high- or medium-use facilities, low-use facilities tended to serve older patients (ie, ≥85 years: 2% and 23% vs 28%) and those living in nonmetropolitan areas (21% and 22% vs 39%) (Table 3).

**Figure.**
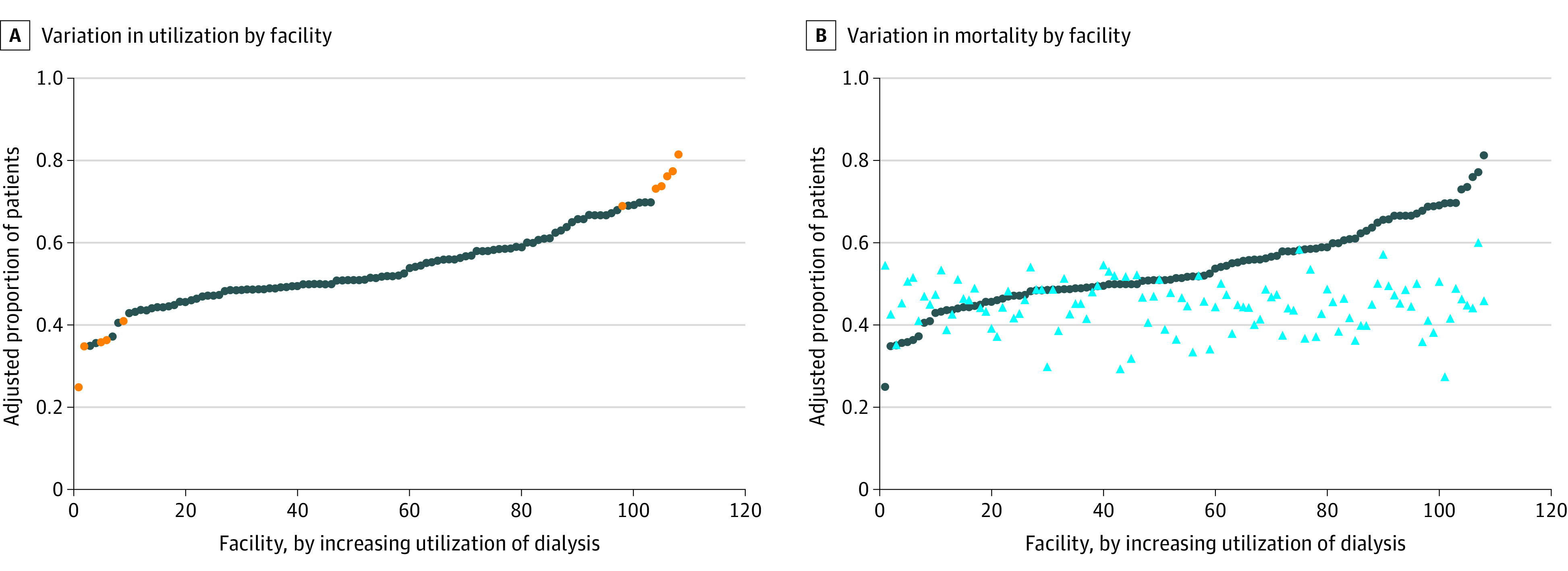
Variation in Dialysis Use and Mortality by Facility Blue circles in each panel represent the facility proportion of patients with kidney failure who received dialysis, adjusted for patient characteristics. A, Orange circles indicate that a facility’s dialysis use is statistically significantly different from the mean (0.54). B, Light blue triangles indicate the facility proportion of patients with kidney failure who died, adjusted for patient characteristics. The correlation coefficient between dialysis use and mortality was 0.03.

**Table 3.** Facility and Patient Characteristics by Facility Percentile of Dialysis Use^a^

Variable	No. (%)			*P* value
	<10th percentile	10th to 90th percentile	>90th percentile	
**Facility characteristics**				
No.	10	88	10	NA
High complexity	5 (50)	63 (72)	6 (60)	.31
VISN region				
Northeast	2 (20)	16 (18)	2 (20)	>.99
West	3 (30)	20 (23)	1 (10)	
Southeast	3 (30)	31 (35)	4 (40)	
Midwest	2 (20)	21 (24)	3 (30)	
VA outpatient dialysis unit	6 (60)	53 (60)	6 (60)	.97
Dialysis unit maximum capacity, mean (SD)	55.3 (26.7)	58.5 (29.1)	49.8 (32.2)	.87
Dialysis unit, % occupancy, mean (SD)	91.1 (6.1)	85.2 (28.9)	87.0 (15.0)	.98
**Patient characteristics**				
No.	678	7312	705	NA
Age group, y				
67 to <70	92 (14)	1196 (16)	118 (17)	.04
70 to <75	129 (19)	1407 (19)	125 (18)	
75 to <80	125 (18)	1489 (20)	135 (19)	
80 to <85	144 (21)	1524 (21)	174 (25)	
≥85	188 (28)	1696 (23)	153 (2)	
Female sex	9 (1)	110 (2)	3 (0)	.07
Race/ethnicity				
White	512 (76)	5017 (69)	573 (81)	<.001
Black	134 (20)	1630 (22)	89 (13)	
Other^b^	31 (5)	658 (9)	42 (6)	
Missing	1 (0)	7 (0)	1 (0)	
Residence				
Metropolitan	413 (61)	5713 (78)	559 (80)	<.001
Nonmetropolitan	262 (39)	1599 (22)	146 (21)	
Zip code median income, US $				
<41 500	164 (24)	2540 (35)	234 (33)	<.001
41 500 to <54 000	228 (34)	2173 (30)	301 (43)	
≥54 000	270 (40)	2503 (34)	160 (23)	
Missing	16 (2)	96 (1)	10 (1)	
Driving distance to nearest VA facility, miles	32.8 (13.3 to 64.4)	26.0 (11.0 to 58.4)	44.5 (19.9 to 92.7)	<.001
Co-payment for VA services	498 (74)	5583 (76)	539 (77)	.18
Comorbidities				
Diabetes	415 (61)	4567 (63)	428 (61)	.56
Ischemic heart disease	320 (47)	3562 (49)	370 (53)	.11
Peripheral vascular disease	218 (32)	2201 (30)	218 (31)	.51
Cerebrovascular disease	168 (25)	1762 (24)	147 (21)	.13
Congestive heart failure	300 (44)	2972 (41)	255 (36)	.01
Cancer	219 (32)	2013 (28)	197 (28)	.03
Chronic lung disease	230 (34)	2516 (34)	229 (33)	.58
Liver disease	41 (6)	394 (5)	32 (5)	.46
Dementia	67 (10)	689 (9)	56 (8)	.38
Paralysis	11 (2)	130 (2)	12 (2)	.95
Rheumatologic disease	20 (3)	174 (2)	24 (3)	.19
Depression/PTSD	166 (25)	1529 (21)	129 (18)	.02
Nursing home resident	78 (12)	663 (9)	46 (7)	.01
Hospitalizations in previous year, median (IQR)	1.0 (0.0 to 2.0)	0.0 (0.0 to 1.0)	0.0 (0.0 to 1.0)	<.001
Serum albumin level, mg/dL				
≥3.5	328 (48)	4041 (55)	362 (51)	<.001
<3.5	275 (41)	2537 (35)	241 (34)	
Missing	74 (11)	734 (10)	102 (15)	
eGFR at incident kidney failure, mL/min/1.73 m^2^				
≥10	581 (86)	6213 (85)	582 (83)	<.001
<10	97 (14)	1051 (14)	110 (16)	
Rate of eGFR decline, median (IQR), mL/min/y	−3.2 (−5.4 to −1.8)	−3.5 (−5.9 to −2.0)	−4.0 (−7.0 to −2.1)	<.001
Pre–kidney failure nephrology visit				
None	279 (41)	2624 (36)	342 (49)	<.001
1	55 (8)	562 (8)	56 (8)	
≥2	344 (51)	4126 (56)	307 (44)	

Abbreviations: eGFR, estimated glomerular filtration rate; IQR, interquartile range; NA, not applicable; PTSD, posttraumatic stress disorder; VA, US Department of Veterans Affairs; VISN, Veterans Integrated Service Network.

SI conversion factors: To convert miles to kilometers, multiply by 1.6; serum albumin to grams per liter, multiply by 10.

^a^ Percentiles were adjusted for patient characteristics using the patient-level model discussed in the Methods. The lowest percentile had less than 43.3% of patients with incident kidney failure start dialysis; the highest percentile had greater than 68.9% of patients with incident kidney failure start dialysis. Residence (metropolitan vs nonmetropolitan) and zip code median income were not selected in the final model.

^b^ Other includes Pacific Islander, Asian, and American Indian.

### Variation in Mortality

The observed frequency of mortality across facilities ranged from 27.2% to 60.0%, with a median (IQR) rate of 45.2% (41.2%-48.6%). The unadjusted MRR for mortality was 1.05, and the fully adjusted MRR, including facility and patient characteristics, was 1.08 (Table 2). Compared with younger individuals, slightly less variation in mortality was found among individuals 80 years or older (MRR, 1.05 vs 1.10). In sensitivity analyses restricted to the cohort with eGFR less than 15 mL/min/1.73 m^2^, the fully adjusted MRR for mortality was unchanged (Table 2). Among individuals of all ages, the fully adjusted VPC for mortality was near 0 (range, 0.002-0.005).

Facilities with higher dialysis use had similar mortality rates as facilities with lower dialysis use (Figure, B). That is, no statistically significant correlation was observed between facility-level dialysis use and overall mortality (correlation coefficient = 0.03).

### Correlates of Patient-Level Dialysis Use and Mortality

Table 4 shows facility and patient characteristics that were associated with dialysis use and mortality at the patient level. Of the facility characteristics examined, only dialysis unit capacity was significantly, albeit marginally, associated with either outcome: the higher the dialysis unit capacity, the lower the mortality rate within 2 years of incident kidney failure (rate ratio [RR], 0.98; 95% CI, 0.96-0.99). Individuals who were older (ie, ≥85 years: RR, 0.44; 95% CI, 0.40-0.49), female (RR, 0.47; 95% CI, 0.34-0.65), or had a diagnosis of cancer (RR, 0.80; 95% CI, 0.75-0.86) or dementia (RR, 0.60; 95% CI, 0.53-0.69) were less likely to start dialysis. Although Black individuals had a similar likelihood of dialysis use compared with White individuals (RR, 1.05; 95% CI, 0.97-1.14), they were less likely to die within 2 years of incident kidney failure (RR, 0.81; 95% CI, 0.74-0.88). Nephrology care in the year before incident kidney failure was associated with a lower risk of dialysis use (RR, 0.66; 95% CI, 0.62-0.71) and mortality (RR, 0.75; 95% CI, 0.70-0.80) within 2 years.

**Table 4.** Association Between Facility and Patient Characteristics With Patient-Level Dialysis Use and Mortality^a^

Variable	Rate ratio (95% CI)	
	Dialysis use	Mortality
**Facility characteristics**		
Facility complexity		
Medium/low	1 [Reference]	1 [Reference]
High	1.09 (0.89 to 1.33)	1.17 (1.05 to 1.31)
VISN region		
Northeast	1 [Reference]	1 [Reference]
West	0.98 (0.77 to 1.25)	1.06 (0.94 to 1.19)
Southeast	1.07 (0.86 to 1.33)	1.05 (0.94 to 1.17)
Midwest	0.91 (0.72 to 1.15)	0.97 (0.87 to 1.09)
VA outpatient dialysis unit, vs none	0.70 (0.47 to 1.05)	1.09 (0.91 to 1.32)
Dialysis unit maximum capacity, per 10 patients	1.01 (0.98 to 1.05)	0.98 (0.96 to 0.99)
Dialysis unit, % occupancy, per 10%	1.00 (1.00 to 1.00)	1.00 (1.00 to 1.00)
**Patient characteristics**		
Age group, y		
67 to <70	1 [Reference]	1 [Reference]
70 to <75	0.95 (0.87 to 1.04)	1.19 (1.04 to 1.35)
75 to <80	0.85 (0.78 to 0.93)	1.50 (1.32 to 1.71)
80 to <85	0.75 (0.68 to 0.83)	1.98 (1.75 to 2.24)
≥85	0.44 (0.40 to 0.49)	2.83 (2.51 to 3.18)
Female sex	0.47 (0.34 to 0.65)	0.82 (0.62 to 1.09)
Race/ethnicity		
White	1 [Reference]	1 [Reference]
Black	1.05 (0.97 to 1.14)	0.81 (0.74 to 0.88)
Other	0.86 (0.77 to 0.96)	0.85 (0.75 to 0.96)
Driving distance to nearest VA facility, per 10 miles	1.02 (1.01 to 1.03)	1.00 (0.99 to 1.01)
Co-payment for VA services, yes vs no	NA	1.14 (1.05 to 1.23)
Comorbidities		
Diabetes	1.22 (1.15 to 1.31)	NA
Ischemic heart disease	1.14 (1.07 to 1.22)	1.05 (0.98 to 1.12)
Peripheral vascular disease	1.15 (1.07 to 1.22)	1.19 (1.11 to 1.28)
Cerebrovascular disease	NA	1.14 (1.06 to 1.22)
Congestive heart failure	1.11 (1.04 to 1.18)	1.36 (1.27 to 1.46)
Cancer	0.80 (0.75 to 0.86)	1.21 (1.12 to 1.29)
Chronic lung disease	1.06 (0.99 to 1.13)	1.17 (1.09 to 1.26)
Liver disease	1.14 (1.01 to 1.30)	1.35 (1.18 to 1.54)
Dementia	0.60 (0.53 to 0.69)	1.38 (1.25 to 1.53)
Depression/PTSD	NA	NA
Paralysis	NA	1.11 (0.88 to 1.40)
Rheumatologic disease	NA	0.99 (0.81 to 1.20)
Nursing home resident	NA	1.10 (0.99 to 1.22)
Hospitalizations in the previous year, per 1-unit increase	NA	1.07 (1.04 to 1.10)
Serum albumin level, per 1 mg/dL	0.57 (0.53 to 0.62)	0.67 (0.63 to 0.72)
eGFR at incident kidney failure, mL/min/1.73 m^2^		
<10	1 [Reference]	1 [Reference]
≥10	0.46 (0.43 to 0.50)	0.83 (0.76 to 0.91)
Rate of eGFR decline, per 5 mL/min/y	1.00 (0.99 to 1.01)	0.99 (0.99 to 1.00)
Pre–kidney failure nephrology visit, vs none	0.66 (0.62 to 0.71)	0.75 (0.70 to 0.80)

Abbreviations: eGFR, estimated glomerular filtration rate; NA, not applicable; PTSD, posttraumatic stress disorder; VA, US Department of Veterans Affairs; VISN, Veterans Integrated Service Network.

SI conversion factors: To convert miles to kilometers, multiply by 1.6; serum albumin to grams per liter, multiply by 10.

^a^ Variable selection was performed to identify the patient characteristics that were included in each model. Some variables (eg, co-payment) were selected for inclusion in the model for dialysis use and not in the model for mortality.

## Discussion

In the VA health care system, we found sizable unexplained variation in dialysis use practices across facilities after accounting for a number of patient and facility characteristics. The magnitude of between-facility variation in dialysis use was larger than that for mortality, suggesting that these variations were clinically meaningful. Furthermore, the pattern of dialysis use across facilities did not appear to correlate with global differences in how sick patients were, as measured by facility mortality rate.

Variation in health care utilization is frequently observed in aspects of health care in which consensus treatment practices are lacking. We found a 1.4-fold variation in rates of dialysis use across VA facilities after accounting for a range of patient and facility characteristics. The magnitude of between-facility variation in dialysis use was similar to the effect sizes for clinical characteristics such as cancer, dementia, and pre–kidney failure nephrology care; and the magnitude was smaller than the associations of age of 85 years or older, female sex, and serum albumin level. Variation in the practice of dialysis use among patients with eGFR of 15 mL/min/1.73 m^2^ or greater (so-called early start) accounted for some of the differences in dialysis use patterns.

On one hand, given the open-ended nature of the guidelines on appropriate use of dialysis for older adults with kidney failure, this degree of variation may be unsurprising. The degree of variation we observed for dialysis was similar to that found in more consensus-driven practices, such as the prescription of renin-angiotensin-aldosterone system blockers for adults with CKD and diabetes.^18^ On the other hand, it is striking that the use of a life-sustaining treatment varies to a similar extent as the use of preventive treatments. Unexplained variation in dialysis use was more strongly associated with patient characteristics than facility characteristics, which could reflect strong institutional norms or unmeasured differences in clinician practices.^19,20,21^ In the present cohort study, we could not directly assess patient preferences, although studies outside the VA system have suggested it is unlikely that preferences would vary to this degree across facilities.^22^

Facilities with less aggressive patterns of dialysis use had similar mortality outcomes compared with those with more aggressive patterns of dialysis use, a finding that was consistent with previous research that found that lower dialysis use among patients treated in VA facilities vs under Medicare was not associated with worse overall survival.^6^ The transition from advanced CKD to kidney failure is associated with intensive health care utilization and high mortality rates, particularly among those with high comorbidity burden.^23^ Although guidelines highlight the uncertain benefits of dialysis in this population, qualitative research suggests that dialysis is viewed as the standard of care in some settings, with strong system-level forces that impede the ability of patients to opt out of this treatment.^24^ The findings from this study underscore the need for greater equipoise in these settings and highlight potential opportunities for improvement in care delivery, such as routine elicitation and documentation of patient preferences as well as explicit efforts to establish alternative models of care and to normalize nondialytic supportive care as a treatment option. Coordinated policy efforts may be needed to ensure that patient preferences are not overruled by clinical momentum and that dialysis treatment is not provided by default to patients who prefer supportive care but have difficulty accessing these programs. Differences in the availability and organization of supportive care services across facilities may exist that we were not able to ascertain.^25^ For example, nutritional counseling to delay the appearance of uremic symptoms as well as coordinated palliative care and social services have been described as important components of kidney supportive care programs in other health care systems.^26^

The absence of statistically significant variation in total mortality rates among older adults with kidney failure served by the VA system contrasts with other work such as the Dialysis Outcomes and Practice Patterns Study, which has found marked variation in mortality after dialysis initiation, leading many to speculate that dialysis selection and initiation practices may underlie this variation.^8^ A potential reason for this difference is that the VA health care system is an organization that is structured relatively uniformly and serves a more homogeneous patient population compared with fee-for-service settings. In addition, in integrated health care systems such as the VA, no financial incentive exists to use dialysis treatment when it is not aligned with an individual’s goals.

In this cohort of older adults with kidney failure, we found that variation in dialysis use and mortality was more strongly associated with between-patient differences than with between-facility differences. Consistent with past studies, in the present study, increasing age was associated with lower rates of dialysis use and higher mortality rates, and Black race/ethnicity was associated with a survival benefit.^27,28,29^ Although individuals who received nephrology care before kidney failure had lower mortality rates, they were less likely to receive dialysis. This finding may be associated with a higher quality of kidney disease–specific care provided by nephrologists, although conflicting evidence of this theory is offered in the literature, which has associated nephrology care with increased risk of kidney disease progression.^30^ In addition, individuals who receive nephrology care may be more compliant with treatment and may have better access to needed treatments and services, thus decreasing their rate of disease progression. These individuals may also have a social support system that allows for travel to and from appointments and fosters the ability to make informed decisions regarding dialysis.

To our knowledge, this study is the first to quantify the association of facility and patient characteristics with variations in dialysis use and mortality among adults with incident kidney failure in the US. By using data from the VA health system, the largest integrated health care system in the US, we were able to evaluate the treatment patterns and outcomes among older adults with incident kidney failure on a national level.

### Limitations

This study has several limitations. The results are specific to veterans and may not be generalizable to other populations, including those who receive care in other health care systems. However, confining our analyses to a system without much variation in ability to pay or financial incentives allowed us to concentrate on other correlates of patient or facility variation. We also lacked information on some facility characteristics, such as staffing and clinician experience, and some patient characteristics, such as family support and housing, that could be associated with dialysis use and mortality. Similarly, we lacked information about patient care preferences.

## Conclusions

We found sizeable unexplained variation in dialysis use practices for older adults across VA facilities that did not appear to be correlated with how sick the patients were. Findings from this study of an integrated system could serve as a benchmark for future studies of the fee-for-service setting and may present opportunities to improve the degree to which dialysis use and initiation practices support the values, goals, and preferences of older individuals with kidney failure.

## Notes

Supplement. **eFigure.** Flow Diagram of Analytic Cohort
