# Supplementary material for: Facility-Level Variation in Dialysis Use and Mortality Among Older Veterans With Incident Kidney Failure
Source: JAMA Netw Open. 2021 Jan 15;4(1):e2034084. doi: 10.1001/jamanetworkopen.2020.34084 (PMC7811178; doi:10.1001/jamanetworkopen.2020.34084)
Supplement: Supplement. — eFigure. Flow Diagram of Analytic Cohort [file jamanetwopen-e2034084-s001.pdf]

Supplementary Online Content

Bradshaw C, Thomas IC, Montez-Rath ME, et al. Facility-level variation in dialysis use and mortality among older veterans with incident kidney failure. *JAMA Netw Open*. 2021;4(1):e2034084. doi:10.1001/jamanetworkopen.2020.34084

eFigure. Flow Diagram of Analytic Cohort

This supplementary material has been provided by the authors to give readers additional information about their work.

©2021 Bradshaw C et al. *JAMA Network Open*

eFigure. Flow Diagram of Analytic Cohort

Abbreviations: eGFR – estimated glomerular filtration rate

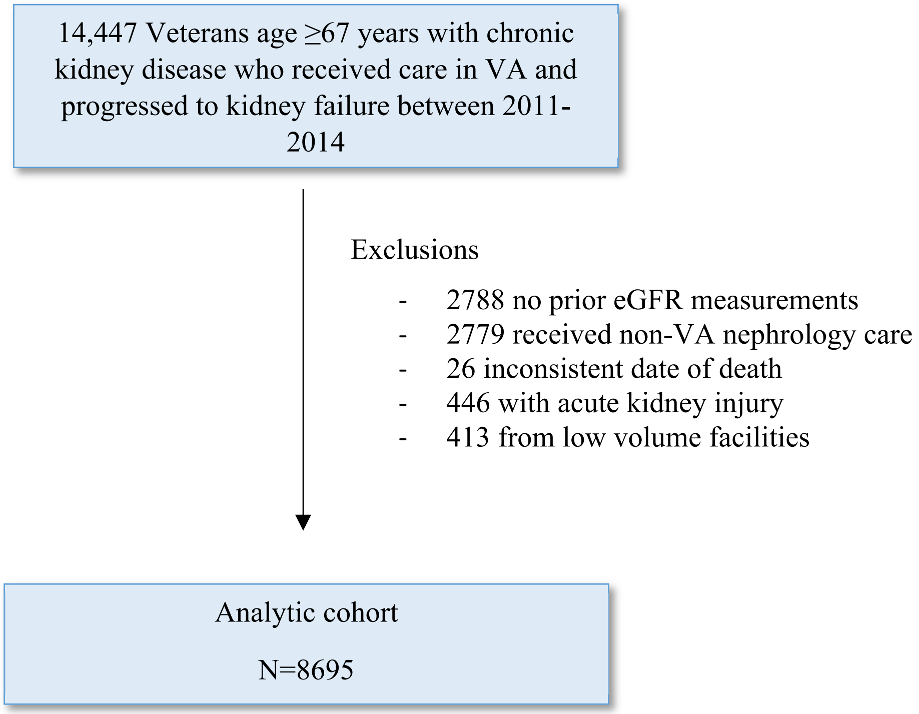

©2021 Bradshaw C et al. *JAMA Network Open*
